# Effects of Dietary Direct Fed Microbial Supplementation on Performance, Intestinal Morphology and Immune Response of Broiler Chickens Challenged With Coccidiosis

**DOI:** 10.3389/fvets.2019.00463

**Published:** 2019-12-12

**Authors:** Ali Calik, Islam I. Omara, Mallory B. White, Wenting Li, Rami A. Dalloul

**Affiliations:** ^1^Avian Immunobiology Laboratory, Department of Animal & Poultry Sciences, Virginia Tech, Blacksburg, VA, United States; ^2^Department of Animal Nutrition & Nutritional Diseases, Faculty of Veterinary Medicine, Ankara University, Ankara, Turkey; ^3^Department of Animal Production, Faculty of Agriculture, Cairo University, Giza, Egypt; ^4^Animal Nutrition, DuPont Nutrition & Biosciences, Wilmington, DE, United States

**Keywords:** broiler, coccidiosis, direct feed microbials, performance, immune response, cytokine

## Abstract

Poultry coccidiosis is a costly intestinal disease that leads to considerable tissue damage, inefficient nutrient absorption, increased mortality, and predisposition to secondary infections. This study evaluated the effects of a direct feed microbial (DFM) dietary additive on performance, intestinal morphology, and immune response of broilers during a mixed coccidiosis challenge. In total, 840 Cobb500 male broilers were randomly allocated to 3 treatments (7 replicates, 40 birds/pen) including negative control (NC) fed basal diet; positive control (PC) fed basal diet with coccidiosis challenge; and DFM supplemented diet, with coccidiosis challenge. At 15 days of age, all birds except for the NC treatment were orally gavaged with live oocysts of a commercial vaccine. On d 21 (6 days post challenge), 4 birds/pen were randomly selected and euthanized for scoring of coccidia-caused lesions in the duodenum, jejunum, and ceca. Body weight gain (BWG), feed intake (FI), and feed conversion ratio (FCR) were recorded on d 7, 14, 28, and 42. Jejunal and ileal tissue samples were taken for histomorphological assessment from 2 birds/pen on d 21. Ileal samples were also taken for mRNA expression analysis on d 14 and d 21. The DFM birds had significantly greater BWG than PC birds during d 0–21 (*P* < 0.05). No differences were observed among the treatment groups in terms of FI and FCR. Dietary DFM supplementation significantly reduced lesion scores in the duodenum and jejunum when compared with PC group (*P* < 0.05). The coccidia challenge significantly reduced (*P* < 0.05) ileal villus height when compared to the non-challenged group on d 21. Conversely, dietary DFM supplementation alleviated the negative effects of coccidiosis by increasing ileal villus area on d 21 (*P* < 0.05). The challenged birds had significantly greater expression of IFN-γ and IL-1β in the ileum on d 21. Based on these findings, dietary DFM supplementation may help restore broiler performance during the starter and early grower periods during coccidiosis, likely by maintaining gut integrity via improving intestinal morphology and also by reducing disease severity as manifested by lower lesion scores.

## Introduction

Avian coccidiosis is an important parasitic disease that leads to considerable intestinal tissue damage, inefficient nutrient absorption, and increased mortality resulting in millions of dollars in economic losses to the world poultry industry every year (1, 2). This disease is caused by several species of *Eimeria* of the phylum Apicomplexa (3), which are ubiquitous pathogens in the environment of poultry farms making it difficult to control (2). However, some of the anticoccidials commonly used to control these pathogens have been under scrutiny (4). Despite the treatment and prevention ability of these chemotherapeutic agents against intestinal diseases, increased public concerns over potential drug residues in poultry products and the emergence of drug-resistant pathogens have put restrictions on the use of certain agents (2, 5). Therefore, there is an increasing demand in the poultry industry for new alternative strategies to improve performance and disease resistance including means of establishing a favorable gut microbiota.

Direct fed microbials (DFMs), also known as probiotics, influence the host's health by maintaining balanced gut microbiota, preventing the growth of pathogenic microorganisms, promoting intake and digestion of feed, and enhancing the immune system (6, 7). Dietary use of DFMs significantly influenced broiler performance (8–10), intestinal morphology (11, 12), and the colonization of beneficial microorganisms in the intestine (13). In addition, DFMs are also found to be suitable for chickens to reduce pathogen colonization and invasion in the intestinal tract to prevent several enteric infections such as *Salmonella* Enteritidis (14), *E*. *coli* (15), and *Clostridium perfringens* (16). Among the DFMs, *Bacillus*-based products have become more popular for potential use in broiler diets as alternatives to antibiotic growth promoters to improve both performance and health (17). Due to their spore-forming ability, these bacteria can withstand harsh environmental conditions including during feed processing and pelleting, as well as survive and germinate under conditions of the gastrointestinal tract (18, 19). *Bacillus amyloliquefaciens* is a spore-forming probiotic bacterium that produces a variety of extracellular enzymes including α-amylases, proteases, and phytase which could improve digestion and absorption of certain nutrients. Studies with *Bacillus amyloliquefaciens* have reported improved growth performance and villus morphology (20), modified cecal microbiota and metabolites (17), and increased serum IgG and IgA concentrations of healthy broilers (21). Li et al. (22) suggested that dietary supplementation of *Bacillus amyloliquefaciens* downregulated mRNA abundance of TLR-4, INF- γ, and IL-1β, and improved intestinal barrier junction in LPS-challenged broilers. Moreover, dietary *Bacillus subtilis*-based DFMs reduced the severity of coccidiosis challenge and improved the immune response in broilers (23). Similarly, a recent study showed that *Bacillus amyloliquefaciens* administration reduced coccidial symptoms as evidenced by reduced intestinal lesions and improved villus height (24).

Based on previous findings that suggest the benefits of dietary *Bacillus*-based DFM administration, the current study hypothesized that dietary supplementation of three strains of *Bacillus amyloliquefaciens* may be an effective method to maintain broiler performance and health by influencing intestinal morphology and immune system of broiler chickens during a coccidiosis challenge.

## Materials and Methods

### Birds, Diet and Management

This project was approved and conducted under the guidelines of the Virginia Tech Institutional Animal Care and Use Committee. On day of hatch, 840 male Cobb500 broiler chicks were acquired from a commercial hatchery and transported to the Virginia Tech research facilities. The birds were randomly allocated to three experimental groups each comprising 7 replicate floor pens with 40 birds per pen raised to 42 days (d). The three treatment groups were (1) negative control (**NC**) fed a basal diet without challenge, (2) positive control (**PC**) fed basal diet with coccidiosis challenge, and (3) direct feed microbial (**DFM)**-supplemented basal diet with coccidiosis challenge. The DFM (Enviva® PRO, Animal Nutrition, DuPont Nutrition & Biosciences, DE, USA) consists of three strains of *Bacillus amyloliquefaciens* at 1:1:1 ratio and added to provide 1.5 × 10^5^ CFU/g of feed. The birds had *ad libitum* access to water and a non-medicated corn/soybean-based starter diet (d 0–14) in mash form, and grower (d 15–28) and finisher (d 29–42) in pellet form. All diets were formulated to meet or exceed National Research Council nutrient recommendations (25). Birds were housed in a controlled environment with the ambient temperature thermostatically controlled and gradually reduced from 34°C on the first day to 22°C at 3 weeks, then maintained at 22°C thereafter. The light cycle was 20 h light and 4 h dark throughout the experimental period.

### Coccidia Challenge and Lesion Scoring

At 15 days of age, all birds except for the NC group (which were given 1 mL sterile water) were orally gavaged with 10X the commercial vaccine Advent® (1 mL per bird) containing live oocysts of *Eimeria acervulina, E*. *maxima*, and *E*. *tenella* as previously described by Ritzi et al. (4). On d 21 (6 days post challenge), 28 birds per treatment (4 birds/pen with average pen weight) were randomly selected and euthanized for scoring of coccidia-induced lesions in the duodenum, jejunum, and ceca by personnel blinded to the treatments. Scoring was performed according to the method of Johnson and Reid (26) based on scores ranging from 0 (no gross lesions) to 4 (most severe lesions).

### Birds Growth Performance

Body weight (BW) and feed intake (FI) were recorded on per pen basis at d 14, 21 28, and 42. Body weight gain (BWG) was then calculated for the three feeding phases as well as at the end of the challenge period (d 21). Daily bird mortality and weights were recorded and feed conversion ratios (FCR) corrected accordingly.

### Intestinal Histomorphology

Jejunal (10 cm distal from the bile duct) and ileal (10 cm proximal to the ileocecal junction) tissue samples were taken from 2 birds/pen on d 21. Histological samples were rinsed with ice-cold PBS, preserved in 10% neutral buffered formalin, and shipped to Histo-Scientific Research Laboratories (HSRL, Inc., Mt. Jackson, VA, USA) for slide preparation. The intestinal samples were embedded in paraffin and serially cut into 5 μm sections. Four sections from each jejunum and ileum were mounted on each slide, which were stained using routine procedures for hematoxylin and eosin. Histological measurements and calculations including villus length, mid-point villus width, crypt depth, villus area, and villus height to crypt depth ratio were performed using an Olympus BX50 microscope and SigmaScan Pro 5 software (Olympus America, Melville, NJ, USA) as previously described (27).

### Gut Tensile Strength

On d 22, one bird from each pen (21 birds/treatment) was selected with body weight close to pen average, weighed, euthanized and samples collected to assess gut strength. Sections of the jejunum and ileum were excised, rinsed with sterile PBS, and immediately tested for tensile strength using an Instron Universal Materials Testing Machine (Instron Corp., Norwood, MA, USA) (28).

### Total RNA Extraction and Reverse Transcription

Intestinal tissue samples were taken from 2 birds/pen on each d 14 and d 21 (from birds with average pen weight). Immediately following euthanasia by cervical dislocation, sections were aseptically excised, rinsed in cold PBS, minced on ice-cold surface, snap-frozen in liquid nitrogen, and stored at −80°C. Total RNA was extracted with Trizol reagent following the manufacturer's instructions (ZYMO Research, Direct-zol RNA MiniPrep). Total RNA concentration was determined at optical density (OD) 260 (NanoDrop-1000, Thermo Fisher Scientific, Waltham, MA, USA), and RNA purity was verified by evaluating the ratio of OD 260 to OD 280. After extraction, 2 μg of total RNA were reverse-transcribed into cDNA using the high capacity cDNA Reverse Transcription kit (Applied Biosystems, Carlsbad, CA) following the manufacturer's protocol, and the cDNA was stored at −20°C.

### Quantitative Real-Time PCR

Quantitative real-time PCR (qRT-PCR) was performed using an ABI 7500 Fast Real-Time PCR System (Applied Biosystems). The cDNA was diluted 1:30 in nuclease-free water, and 1 μL of the diluted cDNA was added to each well of a 96-well plate. Next, 9 μL of real-time PCR master mix containing 5 μL of Fast SYBR Green Master Mix (Applied Biosystems), 0.5 μL each of 2 μM forward and reverse primers, and 3 μL of sterile nuclease-free water per reaction were added to each well for a final volume of 10 μL. During the PCR reaction, samples were subjected to an initial denaturation phase at 95°C for 20 s followed by 40 cycles of denaturation at 95°C for 3 s and annealing and extension at 60°C for 30 s. mRNA expression for interferon (IFN)-γ and interleukin (IL)-1β was analyzed using glyceraldehyde-3-phosphate dehydrogenase (GAPDH) as an endogenous control. Each reaction was run in duplicate. Primers were designed (Table 1) using the Primer Express 3.0 software (Applied Biosystems). Results from qRT-PCR were analyzed using the 7500 Real-Time PCR software (Applied Biosystems). Average mRNA abundance relative to the GAPDH endogenous control for each sample was calculated using the 2^−ΔΔCt^ method (29).

**Table 1:** Sequences of primer pairs used for amplification of target and reference genes.

**Gene**	**Primer sequence**	**Size**	**Accession no**.
IFN-γ	GCTCCCGATGAACGACTTGATGTAAG ATGCTGAAGAGTTCATTCG	63	NM_205149.1
IL-1β	CCCGCCTTCCGCTACACACGAAGCA CTTCTGGTTGATG	66	XM_015297469.1
GAPDH	CCTAGGATACACAGAGGACCAGGTT GGT GGA GGAATGGCTGTCA	64	NM_204305

*For each gene, the primer sequence for forward (F) and reverse (R) are listed (5′-3′), the amplicon size (bp) and the NCBI Accession number (Acc) used for the primer design*.

### Statistics

All data were subjected to one-way analysis of variance (ANOVA) using SAS (2004). When significant differences were noted, Tukey's test was performed to separate means and significance accepted at *P* ≤ 0.05.

## Results

### Growth Performance

**Figure 1:**
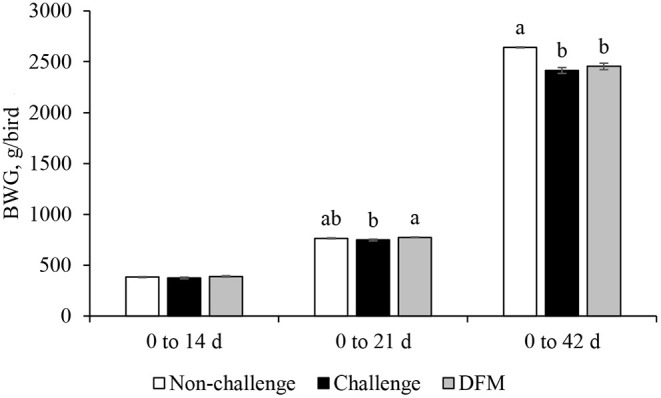
Body weight gain (BWG) of broiler chickens fed control and treatment diets. Each bar represents the mean ± SE (*n* = 7). ^a,b^Bars with different superscripts are significantly different (*P* < 0.05).

**Figure 2:**
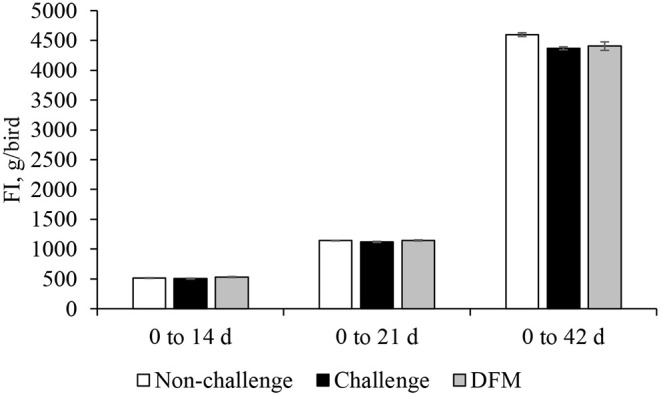
Feed intake (FI) of broiler chickens fed control and treatment diets. Each bar represents the mean ± SE (*n* = 7).

**Figure 3:**
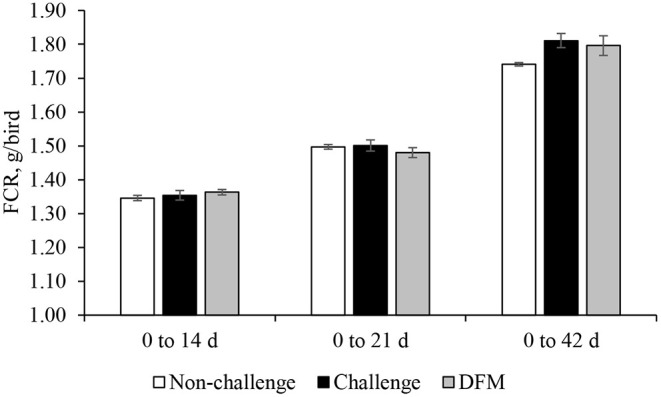
Feed conversion ratio (FCR) of broiler chickens fed control and treatment diets. Each bar represents the mean ± SE (*n* = 7).

Body weight gain (g/bird) is presented in Figure 1. No significant differences in BWG were found between the control groups (non-challenged or challenged) and treatment group from d 0 to d 14. Birds in the DFM group had significantly higher (*P* < 0.05) BWG than the challenged control birds (PC) between d 0 and d 21(NC: 763.5 g; PC: 747.2 g; DFM: 773.7 g). The coccidia challenge reduced the cumulative BWG over the overall experimental period (d 0–42) regardless of dietary DFM supplementation. Feed intake (g/bird) and FCR (g/g bird) are presented in Figures 2, 3, respectively. No significant differences were observed among the treatment groups during d 0–14, d 0–21, and d 0–42, in terms of feed intake and FCR. Although not significantly different, overall mortality rate was slightly higher in challenged (PC: 6.07%, DFM: 6.07%) birds in comparison to non-challenged (4.64%) birds.

### Lesion Scores

**Figure 4:**
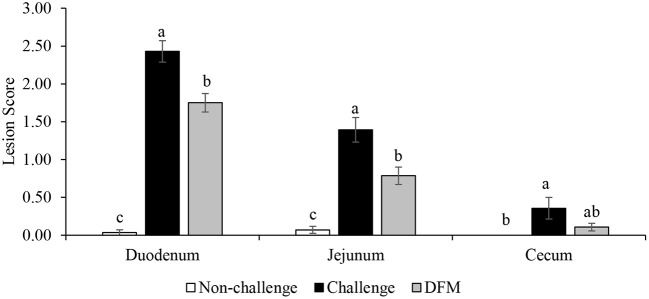
Lesion scores of duodenum, jejunum, and cecum on d 21. ^a–*c*^Bars with different superscripts are significantly different (*P* < 0.05).

Lesion scores are presented in Figure 4. Dietary DFM supplementation significantly reduced lesion scores in the duodenum and jejunum when compared with the PC (challenged) group (*P* < 0.05). As expected, no lesions were observed in the NC (non-challenged) birds.

### Intestinal Histomorphology

Morphological measurements of jejunal and ileal tissues are presented in Table 2. At 6 days post challenge (d 21), jejunum villus height (*P* < 0.05) and villus area (*P* < 0.01) of the birds in the DFM group were significantly greater than birds in the challenge group (PC). Jejunum crypt depths were increased (*P* ≤ 0.001) with the dietary DFM supplementation when compared to no-challenge treatment on d 21. The coccidia challenge significantly reduced (*P* < 0.05) ileal villus height when compared to the non-challenged group on d 21. Conversely, dietary DFM supplementation alleviated the negative effects of coccidiosis by increasing villus area of the ileum on d 21 (*P* < 0.05).

**Table 2:** Effect of dietary DFM supplementation on broiler jejunum and ileum histomorphology 6 days after post challenge (d 21)^1^.

	**Dietary treatments**^****2****^			**Statistics**	
	**Non-challenge**	**Challenge**	**DFM**	**SEM**	***P***
d 21					
Jejunum					
Villus height (μm)	1175^a^^b^	1092^b^	1316^a^	34.14	0.021
Villus width (μm)	153.2	171.7	170.6	4.44	0.164
Crypt depth (μm)	199.0^b^	227.1^a^^b^	253.8^a^	6.78	0.001
VH:CD ratio^3^	6.08	5.07	5.58	0.17	0.057
Villus area (mm^2^)	0.567^b^	0.584^b^	0.713^a^	0.02	0.003
Ileum					
Villus height (μm)	893.2^a^	747.9^b^	834.6^a^^b^	22.35	0.025
Villus width (μm)	131.8^b^	161.7^a^	174.7^a^	4.74	<0.001
Crypt depth (μm)	178.7	192.5	195.5	9.17	0.748
VH:CD ratio	5.30	4.71	4.58	0.16	0.202
Villus area (mm^2^)	0.380^b^	0.3987^b^	0.461^a^	0.02	0.038

a, b *Means with different superscripts in the same row are significantly different (P < 0.05)*.

1 *Data represent mean values of 14 replicates per treatment*.

2 *Non-challenged (negative control (NC) basal diet, without challenge), challenged (positive control (PC), basal diet, with coccidiosis challenge), Direct feed microbial (DFM), DFM supplemented diet, with coccidiosis challenge)*.

3 *Villus height to crypt depth ratio*.

### Gut Tensile Strength

**Figure 5:**
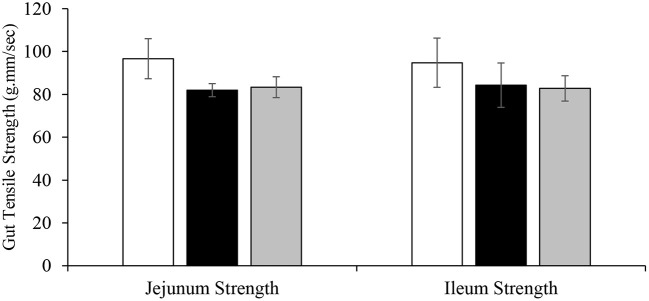
Effect of dietary DFM supplementation on broiler gut tensile strength on d 22. Each bar represents the mean ± SE.

The effect of dietary DFM supplementation on intestinal tensile strength is shown in Figure 5. No significant differences were observed among the treatment groups in terms of intestinal tensile strength on d 22.

### mRNA Expression Analysis

**Figure 6:**
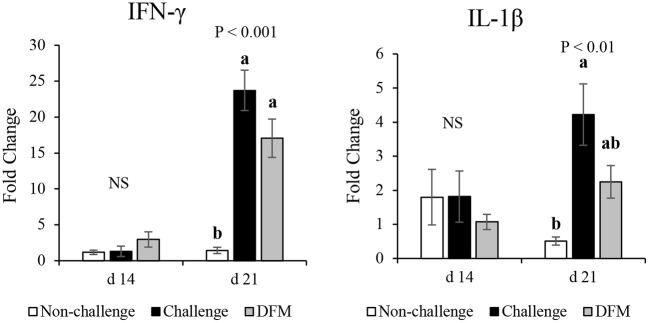
Effect of dietary DFM supplementation on IFN-γ and IL-1β mRNA expression in the ileum on d 21. Each bar represents the mean ± SE. ^a,b^Bars with different superscripts are significantly different (*P* < 0.05). NS, Not significant.

The effect of dietary DFM supplementation on the mRNA expression of IFN-γ and IL-1β in the ileum are shown in Figure 6. Ileal IFN-γ mRNA level was not influenced by dietary treatments before challenge (d 14), but was greater following the coccidia challenge (d 21) regardless of dietary DFM supplementation (*P* < 0.001). The challenge control group (PC) had greater IL-1β mRNA level when compared with the non-challenged (NC) group on d 21 (*P* < 0.01).

## Discussion

Promoting the colonization of beneficial bacteria via non-drug feed additives such as probiotics, to modify the intestinal microbiome and subsequently improve gut barrier function and immune response is becoming an accepted alternative strategy in modern poultry production. However, seeking effective non-drug alternatives to reduce or prevent intestinal pathogens is still under development. In this context, the current work investigated the effects of dietary DFM supplementation on broiler performance, intestinal integrity and immune response of broiler chickens challenged with coccidia.

Coccidiosis is a prevalent intestinal disease characterized by epithelial damage, malabsorption and reduced performance. As an expected outcome, coccidia-challenged birds fed a basal diet displayed retarded growth performance compared with non-challenged control birds. The present study showed that dietary DFM supplementation alleviated the growth suppression effect of coccidiosis by improving BWG at the end of week three when pathology was assessed. Similarly, dietary addition of *Bacillus*-based probiotics restored performance loss compared with coccidia-infected control (no probiotic) birds (23). Moreover, Giannenas et al. (30) noted that birds fed multi-species probiotic mix performed better than infected control birds. The growth promoting effects of DFMs could be related to several modes of action, such as competitive exclusion of pathogens at the epithelial attachment sites, improved intestinal integrity in terms of villi health, or increased concentration of beneficial bacteria in the intestinal tract (31). However, contrary to the observed improvement trend in performance during the grower period, challenged birds, both PC and DFM, had similar BW at the end of the study. These findings are likely due to the compensatory growth potential of fast-growing broilers (32). Further, growth promoting effects of the DFM may be more efficient and pronounced at industrial standards compared to controlled trial as commercial birds are typically exposed to more stressors than a single challenge (33).

Broiler performance and intestinal lesion scores are important parameters used to evaluate the severity of enteric diseases such as coccidiosis (34) and necrotic enteritis (35). *Eimeria* spp. are responsible for mild-to-severe intestinal lesions and these lesions differ across intestinal infection sites depending on the species (3, 36). As expected, no coccidia-induced lesions were found in the intestinal tissues of the non-challenged control birds while coccidial lesions were observed in the challenge control birds without probiotic supplementation (PC). However, birds in the DFM group had less severe duodenal and jejunal lesions compared to PC. These results are in agreement with previous studies in which supplementations of several probiotics were reported to reduce the severity of the intestinal lesions associated with coccidiosis (4, 23). Similarly, Abdelrahman et al. (2) observed significant reduction in oocyst shedding and intestinal lesion scores. Lower lesion scores are indicative of healthier and more functional intestinal epithelium and such changes can be directly correlated with more efficient nutrient utilization and absorption (34, 37). The observed improvements could be attributed to the direct and/or indirect effect of DFMs on colonization and replication of this intracellular parasite in the epithelial tissues, either by competitive exclusion mechanism or possible immune modulatory effects (2, 38). The present study demonstrated that dietary DFM supplementation reduced intestinal lesions under coccidiosis challenge conditions.

Structural changes in the small intestinal architecture can reveal important information about bird performance and gut health (39). Intestinal infections such as coccidiosis, induce villus atrophy (40, 41) and thickening of the lamina propria (41, 42), which retard digestion and nutrient absorption and induce subsequent reduction of growth performance (24). The current findings showed that the coccidiosis challenge significantly influenced jejunal and ileal morphology by decreasing villus height and surface area. Conversely, dietary DFM supplementation alleviated the negative effects of coccidiosis. These results also coincide with reduced intestinal lesion scores. In agreement with the current study, Giannenas et al. (30) concluded that *Bacillus subtilis*-supplemented birds had greater villus height compared to *E*. *tenella* infected birds. Similarly, Tsukahara et al. (24) reported that birds fed a diet containing *Bacillus amyloliquefaciens* (TOA5001) had larger villi than those fed a control diet under a coccidiosis challenge. It is apparent that alimentary- and bacteria-related antigens in the digesta impair the absorptive functions and intestinal integrity (43). However, the use of dietary non-drug alternatives, such as probiotics, may help to maintain intestinal integrity through several possible mechanisms. According to our results, observed improvements in intestinal integrity might be related to the inhibitory effects of DFM against the invasion and proliferation of *Eimeria* parasites. These enhancements also can be attributed to the stimulation of the colonized beneficial microbiota and increased abundance of bacterial metabolites such as butyrate, which could induce enterocyte differentiation and proliferation.

Besides its main function of digestion and absorption, the intestine also plays an important role in protection against pathogens by activation of both adaptive and innate immune responses (44). As an intracellular enteric parasite, *Eimeria* spp. cause epithelial damage and induce inflammation, depending on the severity of the infection, by invading the intestinal mucosa (45). Penetration and invasion of the pathogen triggers a cascade of signaling events that lead to production of various cytokines such as IL-1β, IFN-γ, IL-17, TGF-β, and IL-10 (46, 47). IL-1β and IFN-γ mRNA levels were upregulated in the ileum of the challenged birds, indicating that coccidiosis infection induced inflammation (48). Coccidia-induced upregulation of IFN-γ and/or IL-1β mRNA levels were also documented in previous studies (41, 49–51). Production of these pro-inflammatory Th1 cytokines induces cell mediated immune responses during coccidiosis. As reported herein, no differences were observed between challenge control (PC) and DFM groups in terms of IFN-γ and IL-1β expression levels, except only numerical downregulation in the DFM group compared to PC birds. Dietary use of *Lactobacillus-*based probiotic elevated the intestinal IFN-γ level 3 days post-challenge; however, no differences were observed thereafter (38). This time-dependent difference in the IFN-γ level might be a possible reason in the current study as mRNA levels were evaluated 6 days post-challenge. It should be noted that severity of the infection and the probiotic strain(s) as well as dosage are important considerations when alternative growth stimulators are applied. Therefore, minor differences between PC and DFM birds in terms of IFN-γ and IL-1β mRNA levels might be related to the time of sampling, severity of infection, or probiotic type and dose.

## Conclusion

Based on the presented findings, it can be concluded that dietary DFM supplementation may help restore broiler performance during the starter and grower periods, after coccidian challenge by improving intestinal morphology and reducing lesion severity. Due to its potential beneficial effects on broiler performance and health, this DFM may be used in the broiler industry as a promising alternative when antibiotic growth promoters are not used. Additionally, it would be useful to further assess the effects of this probiotic on specific intestinal tight junction proteins, gut microbiota, and short chain fatty acid composition under similar disease challenge conditions.

## Data Availability Statement

All datasets generated for this study are included in the article/supplementary material.

## Ethics Statement

This project was approved and conducted under the guidelines of the Virginia Tech Institutional Animal Care and Use Committee (VA Tech IACUC).

## Author Contributions

AC, IO, and MW conducted the study and supervised all analyses. AC drafted the manuscript. WL contributed to research design and manuscript revisions. RD was the principal investigator overseeing all aspects of the study. All authors read and approved the final manuscript.

### Conflict of Interest

The authors declare that the research was conducted in the absence of any commercial or financial relationships that could be construed as a potential conflict of interest.
